# Heptagraphene: Tunable Dirac Cones in a Graphitic Structure

**DOI:** 10.1038/srep33220

**Published:** 2016-09-13

**Authors:** Alejandro Lopez-Bezanilla, Ivar Martin, Peter B. Littlewood

**Affiliations:** 1 Argonne National Laboratory, 9700 S. Cass Avenue, Lemont, Illinois, 60439, United States; 2 James Franck Institute, University of Chicago, Chicago, Illinois 60637, United States

## Abstract

We predict the existence and dynamical stability of heptagraphene, a new graphitic structure formed of rings of 10 carbon atoms bridged by carbene groups yielding seven-membered rings. Despite the rectangular unit cell, the band structure is topologically equivalent to that of strongly distorted graphene. Density-functional-theory calculations demonstrate that heptagraphene has Dirac cones on symmetry lines that are robust against biaxial strain but which open a gap under shear. At high deformation values bond reconstructions lead to different electronic band arrangements in dynamically stable configurations. Within a tight-binding framework this richness of the electronic behavior is identified as a direct consequence of the symmetry breaking within the cell which, unlike other graphitic structures, leads to band gap opening. A combined approach of chemical and physical modification of graphene unit cell unfurls the opportunity to design carbon-based systems in which one aims to tune an electronic band gap.

Graphene and its outstanding mechanical and electronic properties lacks one essential feature to be considered as the building block of tomorrow’s electronic devices, namely an electronic band gap. In graphene, as in metallic carbon nanotubes, the conduction and the valence bands touch each other at particular points of the Brillouin zone (BZ) forming the so called Dirac cones, where the energy is directly proportional to the electron momentum. This allows electrons to be considered as massless particles and behave as relativistic charge carriers. The presence of Dirac cones are typically associated to hexagonal lattices (graphene) or structures topologically equivalent. Systems with a lack of hexagonal symmetry exhibiting a Dirac cone are scarce^1^.

In this paper we predict the existence of heptagraphene, a C-based monolayered material exhibiting Dirac cones and formed by the interlock of 7-membered C rings in a rectangular lattice (see Fig. 1). The Dirac cones are demonstrated to be tunable under shearing stress. The structure can be regarded as a massively functionalized graphene, where carbene groups (CH_2_) bridge two opposite atoms in the rings conferring to heptagraphene dynamic stability even at room temperature. It is worth remembering that the reaction of carbenes with organic systems has widely been applied for the synthesis of cyclopropanes and as a non-destructive pathway of attachment of functional groups to the outer walls of carbon nanotubes, while preserving the sidewall hyperconjugation^2^.

As previously observed in carbon nanotubes, a CH_2_ in the proximity of a heptagraphene C-C bond induces a reorientation of the sp^2^ orbitals towards the carbene group C atom, which finally bridges the anchoring atoms leaving their p_*z*_ orbital practically unaltered. Note that although isolated carbene radicals were predicted to easily desorb at room temperature from graphene^3^, we propose a completely different scenario where the carbene groups open the substrate C-C bond and remain chemically attached to the rings forming a periodic pattern. Upon substitution of the H atoms with other functional groups, the CH_2_ can serve as anchoring groups for further chemistry.

The relative complexity of this structure is a challenge for experimental realization but represents a step forward toward the realization of a tunable-gap C-based monolayered material, which is elusive in parent graphitic structures. The use of advanced techniques to embed into the surface foreign species with a small amount of defect-related sites^4^ is proposed as a route for the synthesis of heptagraphene. One of the main goals of this papers is to demonstrate that changing graphene unit cell from hexagonal to rectangular may lead to the creation of Dirac cones and band gap opening upon additional application of shear stress.

## Results

**Figure 1:**
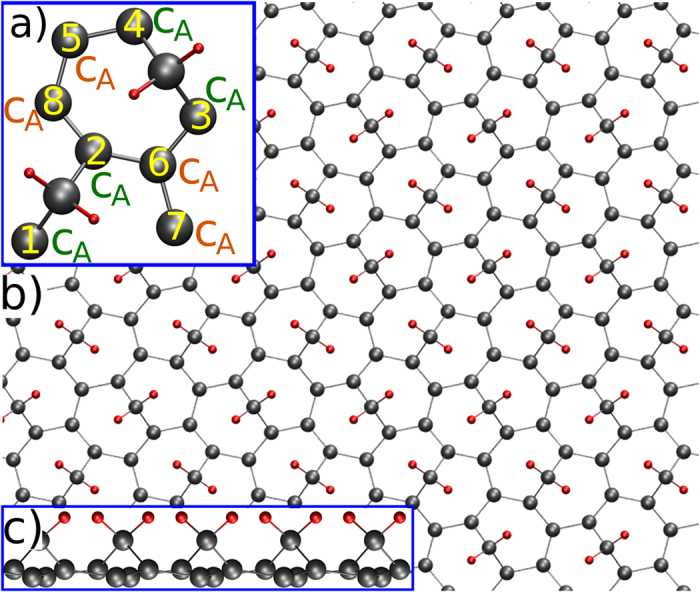
(**a**) 4.78 × 5.70 Å rectangular unit cell of heptagraphene composed of ten atoms of C and four of H. Only the C atoms numbered from 1 to 8 participate in the relevant electronic properties of the structure. (**b**) Top view of the 7-membered ring network of C atoms bridged by CH_2_ groups. (**c**) Side view of the structure where the buckled geometry is recognized. The atoms to which the carbene groups are attached are 0.22 Å above the others.

Figure 1a shows a schematic ball-stick representation of the 4.78 × 5.70 Å edge-rectangular unit cell of heptagraphene containing 10 C and 4 H atoms. Considering the number scheme therein, two types of atoms forming two sublattices can be distinguished: C atoms 1, 2, 3 and 4 (hereafter C_*A*_) are 0.22 Å above the other numbered C atoms (hereafter C_*B*_) (see Fig. 1c). As we will demonstrate, this group division is intimately related to the electronic properties in the vicinity of the Fermi level. According to the division imposed by the CH_2_ groups, the structure can be observed as an arrangement of irregular heptagonal rings (Fig. 1b), hence the name of heptagraphene. Adding up the formation energies of a 8-atom graphene supercell and of two CH_2_ groups, together they are 4.2 eV higher than that of heptagraphene.

After a close inspection of the bond lengths and angles joining neighboring atoms, one concludes that this structure exhibits a noteworthy lack of internal symmetry. The bond length between C atoms are: C_2_-C_8_ = C_8_-C_5_ = C_3_-C_6_ = 1.49 Å, C_5_-C_4_ = C_2_-C_6_ = 1.53 Å. The CH_2_ groups are attached at a distance of 1.51Å to the C_*A*_ atoms forming an angle of 113.1°. Therefore, C atoms anchoring a CH_2_ group own three different bond lengths to each neighbor. Within a hexagonal ring, the angles vary from 107.6° to 138.0° and there are not two equal.

**Figure 2:**
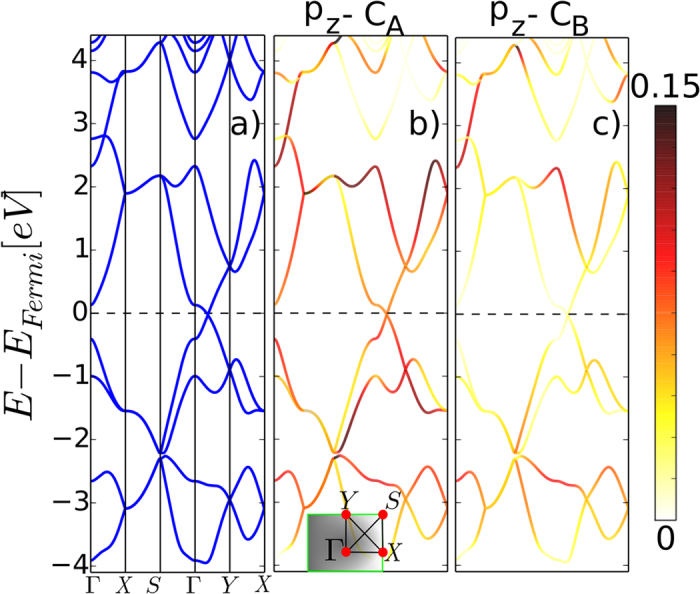
(**a**) Electronic band diagram of heptagraphene. In (**b**,**c**) the color-weighted band diagrams showing the independent contribution to the band diagram in (**a**) of the p_*z*_ orbitals of the atoms C_*A*_ and C_*B*_ in the two sublattices.

According to the geometry of the network and coordination of the C atoms, the predominant orbital hybridization is sp^2^. C_*B*_ atoms deviate ~±5° from the 120° of graphene and only C_*A*_ atoms present stronger distortions in the bonding angles. These deviations from the pure sp^2^ orbital arrangement along with the lack of space-inversion symmetry of the atoms in the unit cell do not prevent the structure from exhibiting a metallic band diagram with a Dirac cone. Figure 2a shows the electronic band diagram of heptagraphene for an energy range of ±4 eV around the Fermi level. A remarkable characteristic is the formation of the Dirac point away from a high-symmetry point, contrary to what commonly occurs in hexagonal lattices. In graphene only under very strong tensile strain a shifted Dirac point has been predicted^5^.

To unravel the contribution of each atomic orbital to the formation of the *π*-bands, color-weighted bands of the p_*z*_ orbitals of the C_*A*_ and C_*B*_ atoms are plotted in Fig. 2b,c respectively. s, p_*x*_, and p_*y*_ orbitals contribute at low energies to the *σ*-bonds, and at high energy levels to the anti-bonding states. Therefore, heptagraphene is eminently a *π* conjugated material, similarly to graphene, although only four out of the eight p_*z*_ orbitals in the unit cell have a significant participation in the formation of the Dirac point. As Fig. 2b shows, solely the C atoms bridged by the carbene groups are responsible of the bands in the vicinity of the Fermi level. The bands spread over a large range of ~5 eV across the BZ. The Dirac cone exhibits a linear dispersion in the Γ to Y direction and becomes flat at the Γ point for the band with opposite slope.

In both bilayer graphene^6^ and silicene^7^ it has been experimentally measured or predicted that the Dirac cone can be precisely controlled at room temperature and with no chemical doping by applying a transverse external electric field. This method is increasingly used in nanostructures with a finite thickness to break inversion symmetry and induce a band gap opening. Since heptagraphene is slightly buckled with one of the two sublattices displaced vertically with respect to the other, one might expect that its electronic structure behaves in a similar manner. Our simulations demonstrate the opposite. The explanation is found in the inequivalent contribution of the two sublattices to the formation to the Dirac cones. Since all the atoms contributing to the electronic bands at the Fermi level (C_*A*_) has the same the same z-component, a band gap opening via electrically controlled sublattice asymmetry cannot be induced.

An appealing strategy towards the modification of the electronic properties of monolayered compounds is to exploit the overlap between neighboring orbitals via tensile or compressive strain. Monolayered materials can stand up against significant tension values and support elastic deformations before rupture, providing the strain engineering a suited technique for tuning their physical properties. In an effort to obtain some benefit from the interplay between the mechanical properties of heptagraphene with its electronic features, stress along several directions were applied. Solely band degeneracy removal and a small change in the slope of the bands forming the Dirac cone was observed upon stretching heptagraphene uni- and bi-axially from −3% up to 5%, demonstrating its robustness against tensile strain. Similarly to graphene, the change in the bond lengths leads to different hopping amplitudes between neighboring orbitals, and as long as the underlying topological structure remains unaltered the band gap remains closed.

**Figure 3:**
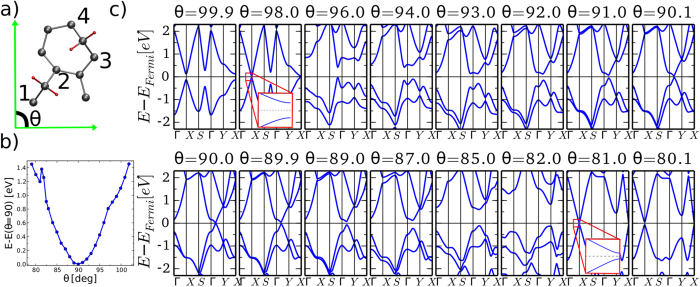
The heptagraphene unit cell is defined by two in-plane lattice vectors forming an angle *θ*, as shown in (**a**). Applying shearing stress modifies *θ* and the total energy of the cell as displayed in (**b**). (**c**) shows the evolution of the electronic bands for increasing and decreasing values of *θ*. The Dirac point observed at *θ* = 90 vanishes for small shearing stress. The insets show that the electronic bands exhibit a blunt ending at the Fermi level.

However, shearing stress was identified as a suitable route to open the heptagraphene band gap. Figure 3 shows the evolution of the electronic bands when the angle *θ* defined by the in-plane unit cell vectors is modified gradually from 90° down to 80.0° and up to 100°. A force acting parallel to one of the cell in-plane vectors is able to break the internal symmetry and induce various types of electronic changes, ranging from the distortion of the Dirac cone to the formation of highly dispersive conic bands at different points of the BZ. The fragility of the Dirac cone is manifested by the gap opening for the smallest shearing tension in both sliding directions.

Before examining in detail the band gap opening, it is worth analyzing the sudden formation of ~1.5 eV dispersive bands at the shearing values of *θ* = 98° and *θ* = 81°. The gradual change of *θ* involves small rearrangements of the atomic coordinates, which are more important in the C_*A*_ atoms due to the absence of a *σ* bond between them. As the *θ* is increased the distance between the C_*A*_ atoms 1 and 2 (as shown in Fig. 3a) decreases, and conversely for the C_*A*_ atoms 3 and 4. The configuration energy of the unit cell evolves in a parabolic fashion as shown in Fig. 3b, where an abrupt change in the slope is observed at *θ* = 98.0°. The explanation for such a transition is the formation of a new *σ*-type chemical bond between atoms 1 and 2 that partially releases the energy accumulated by the strain. As a result, two highly dispersive new bands are formed in the vicinity of the Fermi level. Although both bands nearly close the gap, no Dirac point was observed for any configuration with similar shearing stress. Unlike the bands yielding the Dirac point, the two bands exhibit a blunt ending at the X-point, as show in the inset of Fig. 3c for *θ* = 98.0°.

A similar reasoning can be used to explain the two bands that suddenly reduce the band gap at the X-point for a shearing stress reducing *θ* to 81.0°. The structure recovers locally a hexagonal shape whereas the other two C_*A*_ atoms conserve the CH_2_ group bonded. The evolution of the configuration energy is also parabolic until a new *σ* bond is created between the atoms 3 and 4. From that angle on the curve evolves with a different slope. Note that although the formation of the new bands at the X-point occurs also at a shearing angle of ~9° as in the configuration analyzed above, both the first valence and conduction bands exhibit very different shapes, across the BZ. In this situation where a new C-C bond is reconstructed, the CH_2_ is weakly bonded to the C_*A*_ atoms and, therefore, it could easily desorb the graphitic layer. Figure S1 of Supplementary Information shows the bands diagram of this configuration with only one carbene attached to two C atoms. Two bands nearly touch each other at the Fermi level but at the Y-point.

**Figure 4:**
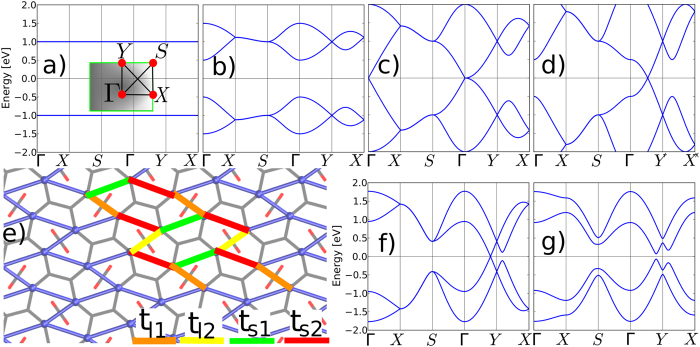
From (**a**–**d**), the electronic band diagram corresponding to a simplified tight-binding model of the geometry shown in (**e**) allows to explain the origin of the shifted Dirac cones. The hopping amplitudes are t_*l*1_ = t_*l*2_ = −1 eV in all four cases. In (**a**), t_*s*1_ = t_*s*2_ = 0 eV, the states of isolated hydrogenic molecules are reproduced. In (**b**), t_*s*1_ = t_*s*2_ = 0.25 eV the localized states interact and some dispersion is developed. In (**c**), t_*s*1_ = t_*s*2_ = 0.5 eV the Dirac point is created. In (**d**), t_*s*1_ = t_*s*2_ = 0.75 eV yields the Dirac point is off-set a high-symmetry line of the Brillouin zone. First-neighbor Hamiltonians derived from a Wannier projection onto the C_*A*_ atoms reproduce the main features of the DFT calculations including the band gap opening, as shown in (**f**,**g**). See main text for numerical values.

The distortion of the Dirac cone leading to band gap opening can satisfactorily be explained within a tight-binding approach. Figure 4e shows the underlying hexagonal network of p_*z*_ orbitals that is topologically equivalent to strained graphene and effectively originates the Dirac cone. This picture can be mapped into a first-neighbor tight-binding Hamiltonian that parametrizes the energetic description of the lattice:
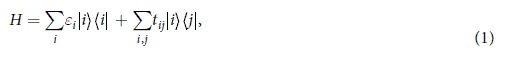
where *ε*_*i*_ is the on-site energy of site *i*, and *t*_*ij*_ is the hopping element to a nearest neighbor site *j* in the C_*A*_ atom based lattice. From our model two types of *t*_*ij*_ are identified, videlicet t_*s*1_ and t_*s*2_ to describe the hopping between two p_*z*_ orbitals in the horizontal zigzagged lines, and t_*l*1_ and t_*l*2_ to describe the hopping between two p_*z*_ orbitals of atoms bridged by a carbene group.

To understand the origin of the shifted Dirac point, we start by analyzing the limit case where t_*s*1_ = t_*s*2_ = 0 and both t_*l*1_ and t_*l*2_ with equal and finite values. Instead of considering an equivalent but oversimplified model such as a brick-wall model, our tight-binding calculations are based on the geometry of the effective hexagonal cell drawn by blue lines in Fig. 4e. In such a case, the system is equivalent to two isolated hydrogenic molecules with two degenerate flat states at ±t_*l*1_ corresponding to the bonding and antibonding states (Fig. 4a). By increasing the hopping values t_*s*1_ = t_*s*2_ the degeneracy is lifted and two dispersive bands are developed at both sides of the Fermi level (Fig. 4b). For t_*s*1_ = t_*s*2_ = −t_*l*1_/2 = −t_*l*2_/2 the bands touch each other at the Γ-point forming a Dirac point (Fig. 4c). As t_*s*1_ = t_*s*2_ increase with respect to t_*l*1_ and t_*l*2_ the Dirac point shifts towards the Y-point and the bands become increasingly dispersive.

In order to describe the changes that shearing stress induces in the nanostructure, we resort to maximally localized Wannier functions (MLWFs)^8^, obtained using the Wannier90 code^9^ from the first-principles ground state, to derive a Hamiltonian description of the system. In particular we focus on a four-band model to reproduce the two valence and two conduction bands in the vicinity of the Fermi level. By projecting onto the p_*z*_ orbitals of the C_*A*_-type atoms and minimizing the MLWF spread, the band structure obtained using the Wannier interpolation method compared to the first-principles calculations was in very good agreement. Due to the extended character of the orbitals and the partial hybridization of some bands with antibonding states of high energy, the projection tends to delocalize the conduction bands, although for the purpose of numerical analysis the Wannier derived Hamiltonian is perfectly valid.

According to the next-neighbor Hamiltonian extracted from the Wannier projection, in the undistorted structure t_*s*1_ = t_*s*2_ = 0.68, and t_*l*1_ = t_*l*2_ = −0.41 eV. The on-site energies are all equal and will be considered hereafter as the zero-energy level. As observed in Fig. 4f, the Dirac cone is created upon crossing of two bands in the Γ-Y line. Breaking the sublattice symmetry by reducing the angle of the in-plane cell vectors in 1°, the hopping values change substantially to t_*s*1_ = 0.63, t_*s*2_ = 0.72, t_*l*1_ = −0.38, and t_*l*2_ = −0.46 eV. This corresponds to a change in the p_*z*_-p_*z*_ bonding distance (represented in hopping amplitudes by t_*l*1_ and t_*l*2_) of ~1%. The on-site energies shift ±0.2 eV for each pair of C_*A*_ sites. Such a small perturbation of the unit cell is enough to open and electronic band gap, as shown in Fig. 4g. Increasing the difference between the on-site energies of the atoms linked by a same carbene group contributes to remove the band degeneracy in the X-S line. This explains the results on shearing stress presented above and clarifies the energy-gap control at the Dirac point by sublattice symmetry breaking.

**Figure 5:**
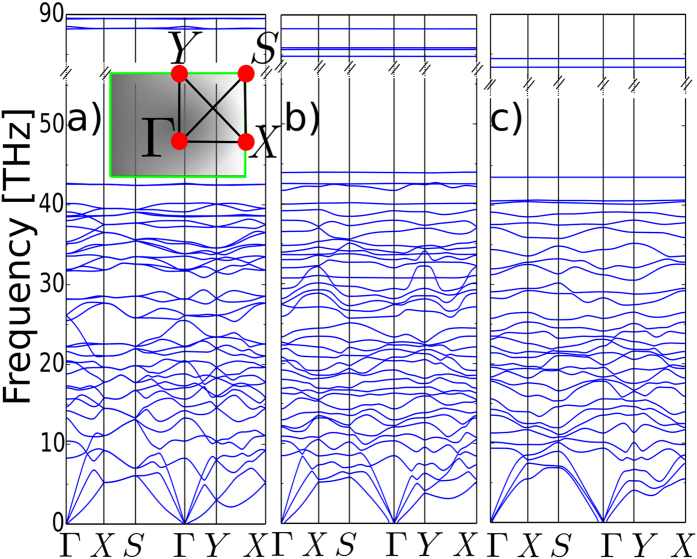
Phonon spectrum of (**a**) heptagraphene, (**b**) sheared heptagraphene *θ* = 81°, (**c**) as in (**b**) but without the CH_2_ group on the reconstructed bond. The frequency (in units of THz) of the phonon modes is plotted versus its propagation direction along the high-symmetry lines Γ → *X* → *S* → Γ → *Y* → *X* of the Brillouin zone, as depicted in the inset.

The dynamical stability of the undistorted monolayer is confirmed by the absence in the phonon spectrum of negative frequencies, as displayed in Fig. 5a along a high symmetry path in the BZ. 42 modes are extended over a large frequency range of 90 THz. The in-plane acoustic branches are characterized by the linear dispersions over most part of the BZ except near the zone edge. The out-of-plane phonon branches exhibit non-linear energy dispersions at both the zone edge and zone center (q = 0). It is notable the energy gap of ~45 THz in the higher half of the spectrum, isolating into a compact energy range at high frequencies a group of four flat branches corresponding to the optical vibrations of the CH_2_ atoms.

There is a limit in the maximum stress that a material can withstand. Beyond that limit the material becomes metastable and long wave perturbations can destroy the structure. The absence of soft modes in the phonon spectrum of the distorted heptagraphene for *θ* = 81° (Fig. 5b) guarantees the dynamic stability of the material. Similarly, for the same stressed configuration but with no CH_2_ group attached to the reconstructed bond (Fig. 5c) is free of instabilities and remains within the elastic limit.

To further verify the robustness of the monolayered structures at finite temperature, DFT-based molecular dynamic simulations with a Nosé thermostat were performed. From heating at 150 K and 300 K a two-dimensional 4 × 4 supercell composed of up to 224 atoms for 5 ps with a time step of 1 fs, no structural rearrangement of the C and H atoms was observed, which further confirms that the nanostructures are stable at room temperature.

Finally, it is worth mentioning that equally interesting results could be attained by chemical decoration with diclorocarbene groups, CCl_2_, another divalent carbon intermediate. Repeating the same type of calculations we observed that the more electronegative Cl atoms reduce the dispersion of the electronic bands. The spread of the bands in the vicinity of the Fermi level is narrowed (see Fig. S2 of Supplementary Information) and the density of electronic states below the Fermi level is enhanced, although the Dirac cone is conserved. This is because the sublattice symmetry is preserved and the renormalization of the hopping matrix elements that define the Dirac cone does not affect them. Only an asymmetric change in the mass of charge carriers due to a flattening of the valence band between Γ and the Dirac point is observed.

## Conclusions

Backed up by detailed calculations of the electronic and mechanical properties of the DFT ground state, we have provided theoretical insight of a new C-based nanostructure formed by ten-membered rings bridged by carbene groups. In heptagraphene every C atom is three-fold coordinated although the C-C angle and bonding distances within the unit cell are anomalously varied, providing the material’s atomic and electronic structure a template to achieve widely tunable band gap configurations in a graphitic system. With only four C atoms participating in the formation of the bands in the vicinity of the Fermi level, an effective structure resembling that of highly strained graphene was found as responsible of the Dirac point in off-set with respect to a high-symmetry point in the BZ. Though the Dirac cones are robust against tensile and compressive strain, we observed large band gap modifications upon application of shearing stress. The Wannier function formalism and the identification of the atomic orbitals involved in the formation of the Dirac cones allowed for a tight-binding analysis that determined the origin of the position and shape of the Dirac cone. The band gap opening under external force application is the result of the internal symmetry breaking as a result of a dimerization of the p_*z*_-p_*z*_ orbital bond strength. Band gap opening for small distortions and bond reconstructions at larger deformation values, accompanied of the creation of different types of dispersive bands can be achieved in dynamically stable configurations. These features of heptagraphene open new possibilities for electronic applications of carbon-based two-dimensional materials and derived nanostructures. We hope that our theoretical results may encourage the experimental synthesis of the material or similar ones with alternative functional groups allowing for the graphene unit cell engineering.

## Methods

First-principles calculations were performed with the generalized gradient approximation to the density functional theory (DFT) and the projector-augmented-wave method as implemented in VASP^10^,^11^,^12^,^13^. The electronic wave functions were computed with plane waves up to a kinetic-energy cutoff of 500 eV. The integration in the k-space was performed using a 20 × 20 × 1 Monkhorst-Pack k-point mesh centered at Γ-point. Lattice constants and atomic coordinates were fully relaxed until the residual forces were smaller than 10^−3^ eV/Å. Phonon calculations were calculated using force-constant method, and the dynamical matrices were computed using the finite differences method in large supercells. The phonon spectra were obtained with the PHONOPY package^14^.

## Additional Information

**How to cite this article**: Lopez-Bezanilla, A. *et al*. Heptagraphene: Tunable Dirac Cones in a Graphitic Structure. *Sci. Rep.* **6**, 33220; doi: 10.1038/srep33220 (2016).

## Supplementary Material

Supplementary Information
