# Supplementary material for: Heptagraphene: Tunable Dirac Cones in a Graphitic Structure
Source: Sci Rep. 2016 Sep 13;6:33220. doi: 10.1038/srep33220 (PMC5020683; doi:10.1038/srep33220)
Supplement: Supplementary Information [file srep33220-s1.pdf]

Heptagraphene: Tunable Dirac Cones in a Graphitic Structure

Alejandro Lopez-Bezanilla[1,*], Ivar Martin[1], and Peter B. Littlewood[1,2]

[1]Argonne National Laboratory, 9700 S. Cass Avenue, Lemont, Illinois, 60439, United States
[2]James Franck Institute, University of Chicago, Chicago, Illinois 60637, United States
[*]alejandrolb@gmail.com

ABSTRACT

Supplementary Information

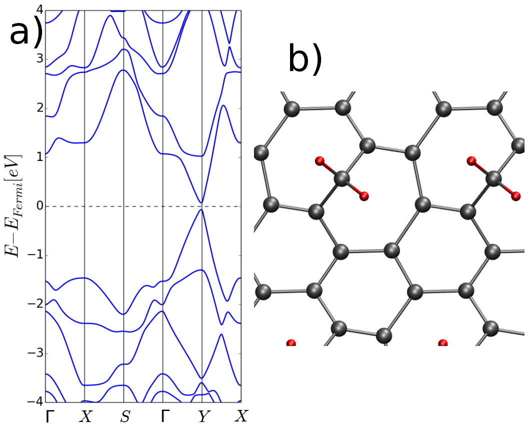

Figure 1. a) Electronic band diagram of heptagraphene with the two in-plane lattice vectors forming an angle of 81° and with no CH_2 group on the reconstructed C-C bond, as shown in b).

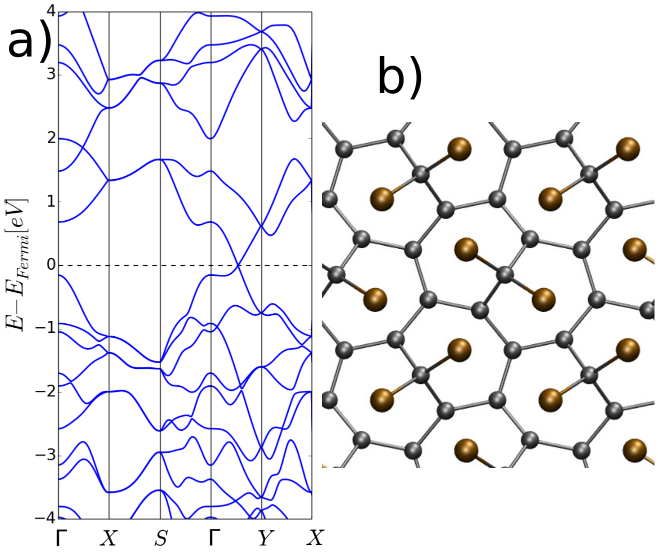

Figure 2. a) Electronic band diagram of heptagraphene with ClH_2 groups, as shown in b).
